# Supplementary material for: Utilization of Healthcare Services in Patients with Chronic Diseases under 18 Years Old: Differences and Contributing Factors
Source: J Pers Med. 2024 Sep 9;14(9):956. doi: 10.3390/jpm14090956 (PMC11433122; doi:10.3390/jpm14090956)
Supplement: Supplementary file 1 [file jpm-14-00956-s001.zip › jpm-3158131-supplementary.pdf]

Journal of Personalized Medicine

MDPI

Table S1. Chronic diseases considered by the Adjusted Morbidity Group (AMG) according to the Strategy for the Care of Chronic Patients of the Community of Madrid.

Alcohol abuse	Human immunodeficiency virus
Anemia	Hyperlipidemia
Aorta aneurysm	Hypertension
Anxiety	Ischemic heart disease
Arthritis	Leukemia
Arthrosis	Liver cancer
Asthma	Lung cancer
Attention–deficit/hyperactivity disorder	Mental retardation
Bladder cancer	Multiple sclerosis
Breast cancer	Obesity
Cardiopulmonary disease	Osteoarthritis
Central nervous system cancer	Osteoporosis
Cervical cancer	Pancreatic cancer
Chronic heart failure	Parkinson
Chronic obstructive pulmonary disease	Prostate cancer
Chronic renal failure	Renal cancer
Cirrhosis	Retinoblastoma
Colon cancer	Schizophrenia
Dementia	Skin cancer
Depression	Soft tissues cancer
Diabetes Mellitus	Stomach cancer
Dysrhythmias	Stroke
Ear, nose and throat cancer	Substance abuse
Endometrial cancer	Testicle cancer
Epilepsy	Thyroid cancer
Gastrointestinal ulcer	Thyroid disorder
Glaucoma	Ulcerative colitis
Hepatoblastoma	Valvular heart disease
Hodgkin/Other lymphomas	Vasculitis

Table S2. Associated severe acute comorbidities considered by the Adjusted Morbidity Group (AMG), because these can be associated with greater complexity.

Central nervous system infection	Pneumonia
Femoral fracture	Pneumothorax
Gangrene	Recurrent urinary infections
Gastrointestinal bleeding	Respiratory failure
Inflammatory bowel disease	Septicaemia
Paralysis	Spinal cord injury
Peritonitis	Tuberculosis

Table S3. Sociodemographic and clinical care characteristics of pediatric patients with chronic diseases users of PC according to sex, age and complexity.

n(%)	Sex		Age				Complexity	
	Female	Male	0–4 years	5–9 years	10–14 years	15–17 years	No	Yes
	169 (45.6)	202 (54.4)	73 (19.7)	114 (30.7)	116 (31.3)	68 (18.3)	357 (96.2)	14 (3.8)
**Sociodemographic**								
Male sex	–	–	35 (47.9)	70 (61.4)	63 (54.3)	34 (50.0)	192 (53.8)	10 (71.4)
Age*	9.3 (5.1)	9.3 (4.5)	2.6 (1.0)	7.0 (1.5)	12.0 (1.4)	16.0 (0.8)	9.5 (4.7)	3.7 (2.7)
Age group 0–4 years	38 (22.5)	35 (17.3)	–	–	–	–	64 (17.9)	9 (64.3)
5–9 years	44 (26.0)	70 (34.7)	–	–	–	–	110 (30.8)	4 (28.6)
10–14 years	53 (31.4)	63 (31.2)	–	–	–	–	115 (32.2)	1 (7.1)
15–17 years	34 (20.1)	34 (16.8)	–	–	–	–	68 (19.0)	0 (0)
Origin Spain	118 (69.8)	146 (72.3)	46 (63.0)	77 (67.5)	81 (69.8)	60 (88.2)	252 (70.6)	12 (85.7)
Rest of Europe	11 (6.5)	10 (5.0)	9 (12.3)	6 (5.3)	5 (4.3)	1 (1.5)	21 (5.9)	0 (0)
Rest of the world	40 (23.7)	46 (22.8)	18 (24.7)	31 (27.2)	30 (25.9)	7 (10.3)	84 (23.5)	2 (14.3)
**Clinical**								
Complexity index*	3.6 (2.3)	3.8 (2.9)	6.0 (3.5)	3.8 (2.4)	3.0 (1.6)	2.3 (1.4)	3.4 (1.9)	12.2 (4.2)
With complexity	4 (2.4)	10 (5.0)	9 (12.3)	4 (3.5)	1 (0.9)	0 (0)	–	–
Chronic diseases*	1.1 (0.4)	1.1 (0.4)	1.1 (0.3)	1.2 (0.4)	1.1 (0.4)	1.2 (0.5)	1.1 (0.3)	1.6 (0.9)
Multimorbidity	16 (9.5)	26 (12.9)	3 (4.1)	15 (13.2)	13 (11.2)	11 (16.2)	37 (10.4)	5 (35.7)

* Measured by mean (standard deviation). PC: Primary Care.

Table S4. Sociodemographic and clinical care characteristics of pediatric patients with chronic diseases users of HC according to sex, age and complexity.

	**Sex**		**Age**				**Complexity**	
**n(%)**	**Female**	**Male**	**0–4 years**	**5–9 years**	**10–14 years**	**15–17 years**	**No**	**Yes**
	56 (47.1)	63 (52.9)	21 (17.6)	43 (36.1)	32 (26.9)	23 (19.3)	116 (97.5)	3 (2.5)
**Sociodemographic**								
Male sex	–	–	8 (38.1)	24 (55.8)	17 (53.1)	14 (60.9)	61 (52.6)	2 (66.7)
Age *	8.6 (4.7)	9.7 (4.6)	2.5 (1.2)	7.1 (1.5)	11.5 (1.4)	16.0 (0.8)	9.4 (4.6)	2.3 (0.6)
Age group 0–4 years	13 (23.2)	8 (12.7)	–	–	–	–	18 (15.5)	3 (100)
5–9 years	19 (33.9)	24 (38.1)	–	–	–	–	43 (37.1)	0 (0)
10–14 years	15 (26.8)	17 (27.0)	–	–	–	–	32 (27.6)	0 (0)
15–17 years	9 (16.1)	14 (22.2)	–	–	–	–	23 (19.8)	0 (0)
Origin Spain	37 (66.1)	50 (79.4)	15 (71.4)	28 (65.1)	24 (75.0)	20 (87.0)	84 (72.4)	3 (100)
Rest of Europe	6 (10.7)	1 (1.6)	2 (9.5)	3 (7.0)	1 (3.1)	1 (4.3)	7 (6.0)	0 (0)
Rest of the world	13 (23.2)	12 (19.0)	4 (19.0)	12 (27.9)	7 (21.9)	2 (8.7)	25 (21.6)	0 (0)
**Clinical**								
Complexity index*	3.7 (2.2)	3.5 (3.3)	6.6 (4.4)	3.5 (2.1)	2.6 (1.4)	2.3 (1.5)	3.3 (2.0)	14.6 (7.9)
With complexity	1 (1.8)	2 (3.2)	3 (14.3)	0 (0)	0 (0)	0 (0)	–	–
Chronic diseases*	1.1 (0.3)	1.1 (0.4)	1.0 (0.0)	1.1 (0.4)	1.1 (0.3)	1.2 (0.4)	1.1 (0.3)	1.0 (0.0)
Multimorbidity	4 (7.1)	9 (14.3)	0 (0)	6 (14.0)	3 (9.4)	4 (17.4)	13 (11.2)	0 (0)

* Measured by mean (standard deviation). HC: Hospital Care.

Table S5. Most prevalent comorbidities in pediatric patients with chronic diseases users of PC according to sex, age, and complexity; Table S6: Most prevalent comorbidities in pediatric patients with chronic diseases users of HC according to sex, age, and complexity.

	Sex		Age				Complexity	
n(%)	Female	Male	0–4 years	5–9 years	10–14 years	15–17 years	No	Yes
	169 (45.6)	202 (54.4)	73 (19.7)	114 (30.7)	116 (31.3)	68 (18.3)	357 (96.2)	14 (3.8)
Asthma	65 (38.5)	81 (40.1)	30 (41.1)	52 (45.6)	42 (36.2)	22 (32.4)	137 (38.4)	9 (64.3)
ADHD	15 (8.9)	25 (12.4)	0 (0)	10 (8.8)	21 (18.1)	9 (13.2)	40 (11.2)	0 (0)
Anaemia	14 (8.3)	24 (11.9)	7 (9.6)	11 (9.6)	15 (12.9)	5 (7.4)	36 (10.1)	2 (14.3)
Pneumonia	20 (11.8)	21 (10.4)	22 (30.1)	12 (10.5)	7 (6.0)	0 (0)	39 (10.9)	2 (14.3)
Obesity	13 (7.7)	20 (9.9)	0 (0)	13 (11.4)	12 (10.3)	8 (11.8)	33 (9.2)	0 (0)
Thyroid disorder	16 (9.5)	9 (4.5)	4 (5.5)	7 (6.1)	8 (6.9)	6 (8.8)	23 (6.4)	2 (14.3)
Hyperlipidaemia	9 (5.3)	14 (6.9)	0 (0)	6 (5.3)	12 (10.3)	5 (7.4)	23 (6.4)	0 (0)
Anxiety	7 (4.1)	11 (5.4)	0 (0)	4 (3.5)	4 (3.4)	10 (14.7)	18 (5.0)	0 (0)
Epilepsy	7 (4.1)	11 (5.4)	5 (6.8)	6 (5.3)	2 (1.7)	5 (7.4)	15 (4.2)	3 (21.4)
Arthritis	5 (3.0)	6 (3.0)	3 (4.1)	3 (2.6)	5 (4.3)	0 (0)	10 (2.8)	1 (7.1)
Recurrent urinary infections	6 (3.6)	3 (1.5)	4 (5.5)	3 (2.6)	2 (1.7)	0 (0)	8 (2.2)	1 (7.1)
Depression	2 (1.2)	4 (2.0)	0 (0)	1 (0.9)	1 (0.9)	4 (5.9)	6 (1.7)	0 (0)
Stroke	3 (1.8)	1 (0.5)	2 (2.7)	1 (0.9)	0 (0)	1 (1.5)	4 (1.1)	0 (0)
Hypertension	0 (0)	2 (1.0)	0 (0)	2 (1.8)	0 (0)	0 (0)	1 (0.3)	1 (7.1)
Cirrhosis	2 (1.2)	0 (0)	2 (2.7)	0 (0)	0 (0)	0 (0)	1 (0.3)	1 (7.1)
Valvular disease	0 (0)	1 (0.5)	1 (1.4)	0 (0)	0 (0)	0 (0)	1 (0.3)	0 (0)
Neoplasia	1 (0.6)	1 (0.5)	2 (2.7)	0 (0)	0 (0)	0 (0)	0 (0)	2 (14.3)
Paralysis	0 (0)	2 (1.0)	1 (1.4)	0 (0)	0 (0)	1 (1.5)	1 (0.3)	1 (7.1)

ADHD: Attention Deficit Hyperactivity Disorder; PC: Primary Care.

Table S6. Most prevalent comorbidities in pediatric patients with chronic diseases users of HC according to sex, age and complexity.

n(%)	Sex		Age				Complexity	
	**Female**	**Male**	**0–4 years**	**5–9 years**	**10–14 years**	**15–17 years**	**No**	**Yes**
	**56 (47.1)**	**63 (52.9)**	**21 (17.6)**	**43 (36.1)**	**32 (26.9)**	**23 (19.3)**	**23 (19.3)**	**3 (2.5)**
Asthma	20 (35.7)	25 (39.7)	9 (42.9)	21 (48.8)	10 (31.3)	5 (21.7)	44 (37.9)	1 (33.3)
ADHD	5 (8.9)	9 (14.3)	0 (0)	4 (9.3)	6 (18.8)	4 (17.4)	14 (12.1)	0 (0)
Anaemia	3 (5.4)	8 (12.7)	1 (4.8)	2 (4.7)	5 (15.6)	3 (13.0)	11 (9.5)	0 (0)
Pneumonia	6 (10.7)	5 (7.9)	5 (23.8)	5 (11.6)	1 (3.1)	0 (0)	11 (9.5)	0 (0)
Obesity	3 (5.4)	5 (7.9)	0 (0)	4 (9.3)	3 (9.4)	1 (4.3)	8 (6.9)	0 (0)
Thyroid disorder	8 (14.3)	5 (7.9)	1 (4.8)	4 (9.3)	4 (12.5)	4 (17.4)	13 (11.2)	0 (0)
Hyperlipidaemia	3 (5.4)	2 (3.2)	0 (0)	1 (2.3)	3 (9.4)	1 (4.3)	5 (4.3)	0 (0)
Anxiety	1 (1.8)	5 (7.9)	0 (0)	1 (2.3)	0 (0)	5 (21.7)	6 (5.2)	0 (0)
Epilepsy	1 (1.8)	5 (7.9)	1 (4.8)	4 (9.3)	1 (3.1)	0 (0)	5 (4.3)	1 (33.3)
Arthritis	4 (7.1)	1 (1.6)	1 (4.8)	2 (4.7)	2 (6.3)	0 (0)	5 (4.3)	0 (0)
Recurrent urinary infections	1 (1.8)	0 (0)	0 (0)	1 (2.3)	0 (0)	0 (0)	1 (0.9)	0 (0)
Depression	0 (0)	0 (0)	0 (0)	0 (0)	0 (0)	0 (0)	0 (0)	0 (0)
Stroke	2 (3.6)	0 (0)	1 (4.8)	0 (0)	0 (0)	1 (4.3)	2 (1.7)	0 (0)
Hypertension	0 (0)	0 (0)	0 (0)	0 (0)	0 (0)	0 (0)	0 (0)	0 (0)
Cirrhosis	0 (0)	0 (0)	0 (0)	0 (0)	0 (0)	0 (0)	0 (0)	0 (0)
Valvular disease	0 (0)	0 (0)	0 (0)	0 (0)	0 (0)	0 (0)	0 (0)	0 (0)
Neoplasia	0 (0)	1 (1.6)	1 (4.8)	0 (0)	0 (0)	0 (0)	0 (0)	1 (33.3)
Paralysis	0 (0)	2 (3.2)	1 (4.8)	0 (0)	0 (0)	1 (4.3)	1 (0.9)	1 (33.3)

ADHD: Attention Deficit Hyperactivity Disorder; HC: Hospital Care.

Table S7. Total use of PC and/or HC services by pediatric patients with chronic diseases according to sex, age and complexity.

n(%)	Sex		Age				Complexity	
	**Female**	**Male**	**0–4 years**	**5–9 years**	**10–14 years**	**15–17 years**	**No**	**Yes**
	194 (45.9)	229 (54.1)	76 (18.0)	129 (30.5)	138 (32.6)	80 (18.9)	408 (96.5)	15 (3.5)
n ≥ 1 (%)	179 (92.3)	207 (90.4)	75 (98.7)	117 (90.7)	125 (90.6)	69 (86.3)	371 (90.9)	15 (100)
M (SD)	9.9 (9.5)	8.5 (7.4)	13.9 (11.1)	9.2 (7.5)	7.4 (6.7)	7.5 (8.0)	8.6 (7.4)	22.7 (18.2)

HC: Hospital Care; M (SD): Mean (Standard Deviation); *n* ≥ 1: number of patients with at least 1 contact; PC: Primary Care.

Table S8. Use of PC services by pediatric patients with chronic diseases according to sex, age and complexity.

n(%)	Sex		Age				Complexity	
	**Female**	**Male**	**0–4 years**	**5–9 years**	**10–14 years**	**15–17 years**	**No**	**Yes**
	194 (45.9)	229 (54.1)	76 (18.0)	129 (30.5)	138 (32.6)	80 (18.9)	408 (96.5)	15 (3.5)
**Total contacts**								
n ≥ 1 (%)	169 (87.1)	202 (88.2)	73 (96.1)	114 (88.4)	116 (84.1)	68 (85.0)	357 (87.5)	14 (93.3)
M (SD)	9.1 (9.4)	7.7 (7.4)	13.2 (11.2)	8.2 (7.2)	6.6 (6.6)	6.8 (8.1)	7.8 (7.3)	22.5 (18.3)
**Contact type**								
Sanitary								
n ≥ 1 (%)	169 (87.1)	202 (88.2)	73 (96.1)	114 (88.4)	116 (84.1)	68 (85.0)	357 (87.5)	14 (93.3)
M (SD)	8.5 (9.0)	7.3 (6.9)	13.0 (11.1)	7.6 (6.4)	6.3 (6.1)	6.2 (7.7)	7.4 (6.8)	21.8 (18.5)
Laboratory								
n ≥ 1 (%)	40 (20.6)	21 (9.2)	8 (10.5)	11 (8.5)	20 (14.5)	22 (27.5)	60 (14.7)	1 (6.7)
M (SD)	0.4 (0.8)	0.1 (0.4)	0.2 (0.7)	0.2 (0.6)	0.2 (0.4)	0.4 (0.8)	0.2 (0.6)	0.1 (0.5)
Administrative								
n ≥ 1 (%)	10 (5.2)	13 (5.7)	0 (0)	10 (7.8)	9 (6.5)	4 (5.0)	22 (5.4)	1 (6.7)
M (SD)	0.2 (1.0)	0.2 (0.8)	0	0.3 (1.4)	0.1 (0.6)	0.1 (0.5)	0.2 (0.8)	0.6 (2.3)
**Contact form**								
Face–to–face								
n ≥ 1 (%)	169 (87.1)	201 (87.8)	72 (94.7)	114 (88.4)	116 (84.1)	68 (85.0)	357 (87.5)	13 (86.7)
M (SD)	8.9 (8.9)	7.5 (7.3)	12.8 (10.2)	8.1 (7.1)	6.6 (6.6)	6.7 (8.1)	7.7 (7.2)	21.3 (15.9)

Telephone								
n ≥ 1 (%)	18 (9.3)	19 (8.3)	14 (18.4)	10 (7.8)	8 (5.8)	5 (6.3)	33 (8.1)	4 (26.7)
M (SD)	0.2 (1.0)	0.1 (0.4)	0.4 (1.6)	0.1 (0.4)	0.1 (0.2)	0.1 (0.2)	0.1 (0.4)	1.1 (3.3)
Home								
n ≥ 1 (%)	1 (0.5)	1 (0.4)	0 (0)	1 (0.8)	0 (0)	1 (1.3)	2 (0.5)	0 (0)
M (SD)	0.01 (0.1)	0.0 (0.1)	0	0.01 (0.1)	0	0.01 (0.1)	0.0 (0.1)	0
**Professional contacted**								
Pediatrician								
n ≥ 1 (%)	137 (70.6)	168 (73.4)	71 (93.4)	111 (86.0)	109 (79.0)	14 (17.5)	292 (71.6)	13 (86.7)
M (SD)	5.3 (7.4)	4.5 (5.2)	10.3 (8.9)	5.2 (4.4)	3.5 (3.4)	1.5 (6.5)	4.4 (5.4)	17.2 (14.0)
Nurse								
n ≥ 1 (%)	137 (70.6)	150 (65.5)	65 (85.5)	82 (63.6)	92 (66.7)	48 (60.0)	273 (66.9)	14 (93.3)
M (SD)	2.0 (2.8)	1.9 (3.0)	2.5 (2.7)	1.8 (3.1)	2.1 (3.3)	1.3 (1.9)	1.8 (2.7)	4.3 (5.9)
Family Physician								
n ≥ 1 (%)	42 (21.6)	39 (17.0)	4 (5.3)	11 (8.5)	15 (10.9)	51 (63.8)	80 (19.6)	1 (6.7)
M (SD)	0.9 (2.3)	0.5 (1.5)	0.1 (0.3)	0.1 (0.4)	0.2 (0.8)	3.0 (3.4)	0.7 (2.0)	0.1 (0.3)

M (SD): Mean (Standard Deviation); *n* ≥ 1: number of patients with at least 1 contact: PC: Primary Care.

Table S9. Use of HC services by pediatric patients with chronic diseases according to sex, age and complexity.

	Sex		Age				Complexity	
***n* (%)**	**Female**	**Male**	**0–4 years**	**5–9 years**	**10–14 years**	**15–17 years**	**No**	**Yes**
	194 (45.9)	229 (54.1)	76 (18.0)	129 (30.5)	138 (32.6)	80 (18.9)	408 (96.5)	15 (3.5)
Total contacts								
n ≥ 1 (%)	56 (28.9)	63 (27.5)	21 (27.6)	43 (33.3)	32 (23.2)	23 (28.8)	116 (28.4)	3 (20.0)
M (SD)	0.8 (2.1)	0.9 (1.9)	0.7 (2.0)	1.0 (2.0)	0.7 (2.2)	0.8 (1.6)	0.9 (2.0)	0.2 (0.4)
External consultations								
n ≥ 1 (%)	56 (28.9)	62 (27.1)	20 (26.3)	43 (33.3)	32 (23.2)	23 (28.8)	116 (28.4)	2 (13.3)
M (SD)	0.8 (1.7)	0.8 (1.9)	0.7 (1.9)	1.0 (2.0)	0.7 (1.8)	0.8 (1.6)	0.8 (1.8)	0.1 (0.4)
Hospitalizations								
n ≥ 1 (%)	15 (7.7)	3 (1.3)	2 (2.6)	2 (1.6)	1 (0.7)	0 (0)	4 (1.0)	1 (6.7)
M (SD)	0.02 (0.2)	0.01 (0.1)	0.03 (0.2)	0.02 (0.1)	0.01 (0.2)	0	0.01 (0.1)	0.1 (0.3)
Hospital day visits								
n ≥ 1 (%)	2 (1.0)	0 (0)	1 (1.3)	0 (0)	1 (0.7)	0 (0)	2 (0.5)	0 (0)
M (SD)	0.04 (0.4)	0	0.01 (0.1)	0	0.04 (0.5)	0	0.02 (0.3)	0

HC: Hospital Care; M (SD): Mean (Standard Deviation); *n* ≥ 1: number of patients with at least 1 contact.

Figure S1. Districts and neighborhoods of Madrid city. 1

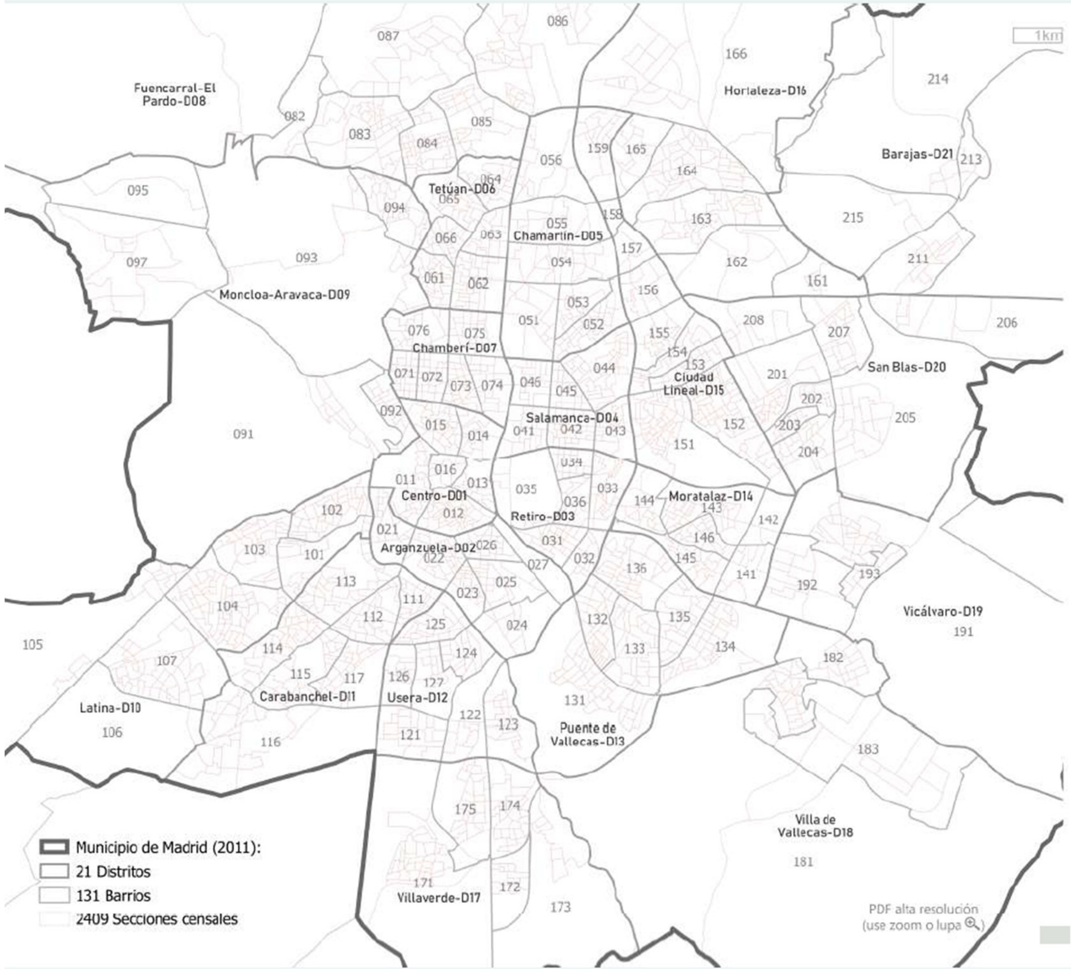

Distrito	nº	Barrio	Distrito	nº	Barrio	Distrito	nº	Barrio
D01	011	Palacio	D08	082	Fuentelarreina	D14	144	Media Legua
D01	012	Embajadores	D08	083	Peña Grande	D14	145	Fontarrón
D01	013	Cortes	D08	084	El Pilar	D14	146	Vinateros
D01	014	Justicia	D08	085	La Paz	D15	151	Ventas
D01	015	Universidad	D08	086	Valverde	D15	152	Pueblo Nuevo
D01	016	Sol	D08	087	Mirasierra	D15	153	Quintana
D02	021	Imperial	D08	088	El Goloso	D15	154	Concepción
D02	022	Las Acacias	D09	091	Casa de Campo	D15	155	San Pascual
D02	023	La Chopera	D09	092	Argüelles	D15	156	San Juan Bautista
D02	024	Legazpi	D09	093	Ciudad Universitaria	D15	157	Colina
D02	025	Delicias	D09	094	Valdezarza	D15	158	Atalaya
D02	026	Palos de Moguer	D09	095	Valdemarín	D15	159	Costillares
D02	027	Atocha	D09	096	El Plantío	D16	161	Palomas
D03	031	Pacífico	D09	097	Aravaca	D16	162	Piovera
D03	032	Adelfas	D10	101	Los Cármenes	D16	163	Canillas
D03	033	Estrella	D10	102	Puerta del Ángel	D16	164	Pinar del Rey
D03	034	Ibiza	D10	103	Lucero	D16	165	Apóstol Santiago
D03	035	Jerónimos	D10	104	Aluche	D16	166	Valdefuentes
D03	036	Niño Jesús	D10	105	Campamento	D17	171	San Andrés
D04	041	Recoletos	D10	106	Cuatro Vientos	D17	172	San Cristóbal
D04	042	Goya	D10	107	Las Águilas	D17	173	Butarque
D04	043	Fuente del Berro	D11	111	Comillas	D17	174	Los Rosales
D04	044	Guindalera	D11	112	Opañel	D17	175	Los Ángeles
D04	045	Lista	D11	113	San Isidro	D18	181	Casco Histórico de Vallecas
D04	046	Castellana	D11	114	Vista Alegre	D18	182	Santa Eugenia
D05	051	El Viso	D11	115	Puerta Bonita	D18	183	Ensanche de Vallecas
D05	052	Prosperidad	D11	116	Buenavista	D19	191	Casco Histórico de
D05	053	Ciudad Jardín	D11	117	Abrantes	D19	192	Valdebernardo
D05	054	Hispanoamérica	D12	121	Orcasitas	D19	193	Valderrivas
D05	055	Nueva España	D12	122	Orcasur	D19	194	El Cañaveral
D05	056	Castilla	D12	123	San Fermín	D20	201	Simancas
D06	061	Bellas Vistas	D12	124	Almendrales	D20	202	Hellín
D06	062	Cuatro Caminos	D12	125	Moscardó	D20	203	Amposta
D06	063	Castillejos	D12	126	Zofío	D20	204	Arcos
D06	064	Almenara	D12	127	Pradolongo	D20	205	Rosas
D06	065	Valdeacederas	D13	131	Entrevías	D20	206	Rejas
D06	066	Berruguete	D13	132	San Diego	D20	207	Canillejas
D07	071	Gaztambide	D13	133	Palomeras Bajas	D20	208	El Salvador
D07	072	Arapiles	D13	134	Palomeras Sureste	D21	211	Alameda de Osuna
D07	073	Trafalgar	D13	135	Portazgo	D21	212	Aeropuerto
D07	074	Almagro	D13	136	Numancia	D21	213	Casco Histórico de Barajas
D07	075	Ríos Rosas	D14	141	Pavones	D21	214	Timón
D07	076	Vallehermoso	D14	142	Horcajo	D21	215	Corralejos
D08	081	El Pardo	D14	143	Marroquina			

2

Source: Gandarillas A, Domínguez MF, Duque I, Cebrecos A, Aránguez E, Ordóñez JM, Cervigón P, Elosegi U. Tercer atlas de mortalidad y desigualdades socioeconómicas en la Comunidad de Madrid, 2008–2015. Consejería de Sanidad, Madrid, 2021. 3 4
